# The determinants of health workers’ job satisfaction in the Saudi Arabian health facilities

**DOI:** 10.1186/s12960-025-01045-9

**Published:** 2026-01-20

**Authors:** Mohannad Alkhateeb, Sayem Ahmed, Solveig Lövestad, Jahangir Khan

**Affiliations:** 1 Department of Health Service and Hospital Administration, Faculty of Economics and Administration, King Abdul Aziz University, Jeddah, 21589 Saudi Arabia; 2 School of Public Health and Community Medicine, Institute of Medicine, Sahlgrenska Academy, University of Gothenburg, Gothenburg, Sweden; 3 Department of Health Sciences, Brunel University London, Uxbridge, United Kingdom; 4 The Västra Götaland Region Competence Centre on Intimate Partner Violence, Gothenburg, Sweden; 5 Department of Learning, Informatics, Management and Ethics, Karolinska Institutet, Stockholm, Sweden; 6 Department of International Public Health, Liverpool School of Tropical Medicine, Liverpool, United Kingdom

**Keywords:** Healthcare workers, Job satisfaction, Determinants, Saudi Arabia, Hospitals

## Abstract

**Background:**

Job satisfaction is closely related to health service quality and patients’ outcomes, including safety and continuity of care. With the transformation of Saudi Arabia’s healthcare system driven by Vision 2030, determining the factors influencing job satisfaction among healthcare workers has become critical.

**Objective:**

To identify the sociodemographic determinants and their association with healthcare workers’ job satisfaction in public hospitals in Jeddah, Saudi Arabia.

**Methods:**

Through a cross-sectional design, healthcare workers from 13 public hospitals were invited to respond to a self-administered questionnaire based on Spector's Job Satisfaction Survey. Data were collected via an electronic online survey (Google Form). Chi-square, ANOVA, independent t-tests, and multiple linear regression analysis were used.

**Results:**

Nine hundred and thirty-two healthcare workers responded. There were significantly more females than males among nurses, midwives, and those with bachelor's qualifications. Conversely, there were significantly more males than females in administrative jobs. Males reported significantly higher income, were more likely to be married, and perform non-clinical work. Higher satisfaction scores were reported among males, non-Saudis, those 50 years or above, holding a diploma education, married, working in a tertiary hospital, and working the day shift. The regression model showed that being female, working in a night or rotating shift were negatively associated with job satisfaction. Being non-Saudi and working part-time were positively associated with job satisfaction.

**Conclusions:**

Job satisfaction was higher among non-Saudis and those working part-time. It was lower among females and those working at night or rotating shift.

## Background

According to Spector, job satisfaction is “the extent to which people like (satisfiers) or dislike (dissatisfiers) their jobs” and is widely adopted in published studies [1]. Job satisfaction among Healthcare Workers (HCWs) is a global challenge that can influence patient safety, care quality, and staff retention [2]. On the other hand, job dissatisfaction can lead to the intention to leave the workplace, which may stop providing quality health services to patients [3]. That is why satisfying HCWs is significant for delivering high-quality services and ensuring continuity of care [4, 5].

Many theories highlight the importance of job satisfaction among workers. Herzberg's two-factor theory, also known as the motivation-hygiene theory, was the earliest to hypothesize the concept of job satisfaction, and it is commonly used in research [6–8]. This theory distinguishes between intrinsic factors that enhance satisfaction and extrinsic factors that prevent dissatisfaction. The extrinsic factors (hygiene) include salary, interpersonal relationships, policies and administration, supervision, and working conditions. While the intrinsic factors (motivation) include advancement in the workplace, work itself, personal growth (promoted in the workplace), responsibility (job-related authority, reflecting trust), recognition, and achievement [9, 10].

Several sociodemographic determinants have been linked to job satisfaction in healthcare workers in Saudi Arabia. However, studies have shown inconsistent relationships among these determinants; for example, while Saudis exhibited higher job satisfaction among dentists [11] and pharmacists [12], the reverse was observed in nurses, where Saudis demonstrated lower job satisfaction than non-Saudis [13]. Similarly, while being male was reported to lead to higher job satisfaction among nurses [13], males exhibited lower satisfaction among pharmacists [12] and physiotherapists [14].

In response to the United Nations’ Sustainable Development Goal 3 (Ensure healthy lives and promote well-being for all at all ages), the Kingdom of Saudi Arabia launched Vision 2030, which was translated into National Transformation Programmes that target good health and well-being and financial efficiency. The programs included crucial initiatives, including Saudization (replacing expatriates with Saudi citizens) as a part of health system privatization. These changes may alter employment contracts, affecting job satisfaction among HCWs, by its negative impact on the job security of non-Saudis [15–17].

The National Transformational Programmes under Vision 2030 started in 2016, shifting from a predominantly government-funded healthcare system to a mixed financing model with the collaboration of the private sector [18, 19]. As a result, the role of the Ministry of Health (MOH) shifted from serving as regulator and provider to only a regulator, and the Health Holding Company (HHC) became the provider of health services through “health clusters.” A health cluster is a group of healthcare facilities in a specific area that work together under one administrative structure and are responsible for the health and wellness of the population [20, 21].

An HHC comprises 1–3 health clusters that include all government hospitals, depending on a region’s size [19]. For example, there were two clusters in Jeddah, one of which included hospitals in the south and the other in the north [22]. The program’s strategy for transforming the health sector included four aspects: better health, better care, better sustainability, and a better workforce through recruiting and training the appropriate number of healthcare professionals and encouraging multidisciplinary teamwork. [16].

One critical challenge facing Saudi Arabia is its dependence on expatriate professionals because of the relative shortage of healthcare staff, exacerbated by an increasing population. For example, expatriate nurses, mainly from India, Malaysia, and the Philippines, constitute 70% of the country’s nursing staff [23, 24]. This complex composition of the HCWs might affect level of JS among both Saudis and non-Saudis, particularly with the progress of Saudization process [25].

This study aimed to identify the sociodemographic determinants and their association with HCWs’ job satisfaction in public hospitals in Jeddah, Saudi Arabia. Understanding the influence of different determinants on job satisfaction among HCWs is crucial for policymakers, as it could help them devise a tailored plan for staff retention and morale building during this transition.

## Method

This cross-sectional study was conducted in Jeddah province, Saudi Arabia. The data were collected between 11 and 31 August 2024 from 13 hospitals in Jeddah, Adham, Rabigh, and Al-Laith, all under the Directorate of Health Affairs in Jeddah province. The study population consisted of all HCWs in the targeted hospitals. This study used Spector’s validated Job Satisfaction survey (JSS) in English and Arabic to measure job satisfaction as the outcome of the study [1].

The questionnaire consisted of two sections. The first section included categorical variables describing the demographic and socioeconomic characteristics of the participants: sex (male and female), nationality (Saudi and non- Saudi), age groups (< 30 years, 30 to < 40 years, 40 to < 50 years, ≥ 50 years), educational levels (diploma, bachelor, and postgraduate), marital status (single, married, divorced and widowed), income (< 5000, 5000 to < 10,000, 10,000 to < 25,000, and ≥ 25,000), years of experience (< 5 years, 5 to < 10 years, 10 to < 15 years, and ≥ 15 years), profession (physician, nurse and midwife, pharmacist, dentist, allied health professional, and administration) as well as relevant variables about hospital type (it is classified depending on location to general urban hospitals, general rural hospitals, and tertiary hospitals), job role (clinical work, non-clinical work, and both), work schedule (day shift, night shift, and day and night shift or rotating shift), work type (full time and part time), job contract (civil services and yearly contract), and current position (staff, managerial, and both).

The second section comprised questions derived from the Job Satisfaction Survey (JSS) by Spector. The JSS is a 36-item scale with nine subscale domains: pay, promotion, supervision, fringe benefits, contingent rewards, operating conditions, coworkers, nature of work, and communication. For example, “I feel I am being paid a fair amount for the work I do” and “I feel unappreciated by the organization when I think about what they pay me” under the pay domain. Each of these nine domains consists of four questions, and each question was assessed by a 6-point scale, ranging from 1 (disagree very much), 2 (disagree moderately), 3 (disagree slightly), 4 (agree slightly), 5 (agree moderately), and to 6 (agree very much). Accordingly, the overall scores ranged between 36 and 216 [1]. The mean score for each domain was obtained by dividing the sum of the responses on each question by 4 (the number of questions). The mean of job satisfaction was used in the regression analysis for prediction using the independent variables.

All HCWs were considered eligible for inclusion in the study. The survey was developed using Google Forms and disseminated via WhatsApp to facilitate efficient and widespread distribution. WhatsApp has been recognized as an effective way of conducting health research, as healthcare workers use it frequently, and it offers ease of access and the ability to reach participants across various locations without disturbing their work routine [26]. Each hospital's Department of Education and Training moderated the dissemination of Google Forms, where all the employees' mobile numbers were available. The research proposal was approved by the institutional review board (IRB) at the Ministry of Health (Directorate of Health Affairs-Jeddah) with reference No. A01874 and the Swedish Ethical Review Authority with reference No. Dnr 2024-02633-01. The study adhered to the ethical principles outlined in the Declaration of Helsinki, ensuring respect for participants’ rights, privacy, and well-being. Informed consent was obtained from the participants in written form on the cover page before starting the questionnaire by ensuring their willingness to be included in the study. The cover page noted that the participants could withdraw anytime without any explanation. The participation was voluntary, and responses were anonymous, so respondents were not traceable. All participants aged 18 years or older, no minors, and no vulnerable groups.

The sample size was calculated using the following formula.

$$n=\frac{{z}^{2}\times \widehat{P}\left(1-\widehat{p}\right)}{{\varepsilon }^{2}}$$

Z-score (Z) of 1.96 corresponding to a 95% confidence level, a margin of error (ε) of 0.05, a population proportion (p̂) of 0.367 (36.7%), which reflects the degree of job satisfaction among healthcare workers as reported in the previous study. Based on these parameters, the required sample size was calculated to be 357 participants [27, 28].

### Data collection

The Health Volunteering website was launched by the Ministry of Health (MOH) in Saudi Arabia in April 2020. The platform is designed to assist researchers by facilitating communication with potential participants, disseminating study materials, and ensuring timely recruitment [29]. It was used to support the data collection process for this study.

The first author coordinated and supervised all stages of data collection. After registering the project on the platform, volunteers were selected by the research and education department. A Zoom orientation meeting was then conducted by the first author to clarify the study objectives, describe the data collection protocol, and outline the volunteers’ specific responsibilities.

The volunteers’ role was limited to distributing the survey link and sending a single reminder message to potential respondents. They were instructed not to influence participants’ responses or collect any identifiable information. All data were self-reported by the participants through an online questionnaire hosted on Google Forms and submitted to the respondents. The system allowed monitoring the number of completed surveys daily to ensure that the targeted sample size was reached within the planned timeframe.

### Statistical analysis

Data were initially entered into an Excel sheet and subsequently transferred to SPSS Version 29 (Statistical Package for the Social Sciences) for analysis. Categorical variables are presented as frequencies and percentages, while quantitative variables (job satisfaction score) are summarised as mean and standard deviation (SD). The Shapiro–Wilk test was performed to determine the normality of the data distribution. A chi-square test was used to determine the significant difference in the categorical variables. The independent sample t-test and one-way ANOVA were conducted to test the significance of the differences in mean job satisfaction scores according to sociodemographic characteristics. A t-test was performed for variables with two categories and one-way ANOVA for three or more categories. Multiple linear regression analysis was conducted to identify the predictors influencing job satisfaction. The regression analysis included sociodemographic variables (sex, nationality, age, educational level, marital status, work schedule, hospital type, years of experience in the profession, monthly income, profession, job role, type of work, job contract, and current position). The *p-*value for significance was < 0.050.

## Results

A total of 932 HCWs from all hospitals completed the questionnaire. Table 1 describes the differences in the characteristics of the study group by sex. It shows significant differences between males and females regarding all displayed characteristics except employment contracts. Females were dominant among non-Saudis (17.5%, *p* < 0.001), nurses and midwives (57.1%, *p* < 0.001), and those holding a bachelor's qualification (60.6%, *p* < 0.001). On the other side, there was a preponderance of males in administrative jobs (19.5%,* p* < 0.001), higher income (69.9%, *p* < 0.001), married (74.7%, *p* < 0.001), and performing non-clinical work (59.7%, *p* < 0.001).

**Table 1:** Sociodemographic characteristics of HCWs by sex in Jeddah province (N = 932)

Variable	Sex			*p* value
	Males, n (%)	Females, n (%)	Both, n (%)	
	435 (46.7)	497 (53.3)	932 (100)	
Nationality				
Saudi	404 (92.9)	410 (82.5)	814 (87.3)	< 0.001
Non-Saudi	31 (7.1)	87 (17.5)	118 (12.7)	
Age categories				
< 30 years	61 (14.0)	72 (14.5)	133 (14.3)	< 0.001
30 to < 40 years	186 (42.8)	282 (56.7)	468 (50.2)	
40 to < 50 years	139 (32.0)	99 (19.9)	238 (25.5)	
≥ 50 years	49 (11.3)	44 (8.9)	93 (10.0)	
Educational level				
Diploma	156 (35.9)	127 (25.6)	283 (30.4)	< 0.001
Bachelor	200 (46.0)	301 (60.6)	501 (53.7)	
Postgraduate	79 (18.2)	69 (13.9)	148 (15.9)	
Marital status				
Single	88 (20.2)	163 (32.8)	251 (26.9)	< 0.001
Married	325 (74.7)	290 (58.4)	615 (66.0)	
Divorced and widowed	22 (5.1)	44 (8.9)	66 (7.1)	
Monthly income (Saudi Riyal)				
< 5000	12 (2.8)	43 (8.7)	55 (5.9)	< 0.001
5000 to < 10,000	101 (23.2)	174 (35.0)	275 (29.5)	
10,000 to < 25,000	304 (69.9)	256 (51.5)	560 (60.1)	
≥ 25,000	18 (4.1)	24 (4.8)	42 (4.5)	
Years of experience in the profession				
< 5 years	79 (18.2)	108 (21.7)	187 (20.1)	< 0.001
5 to < 10 years	76 (17.5)	116 (23.3)	192 (20.6)	
10 to < 15 years	100 (23.0)	138 (27.8)	238 (25.5)	
≥ 15 years	180 (41.4)	135 (27.2)	315 (33.8)	
Profession				
Physician	55 (12.6)	38 (7.6)	93 (10.0)	< 0.001
Nurse and midwife	119 (27.4)	284 (57.1)	403 (43.2)	
Pharmacist	27 (6.2)	6 (1.2)	33 (3.5)	
Dentist	4 (0.9)	10 (2.0)	14 (1.5)	
Allied health professional	145 (33.3)	122 (24.5)	267 (28.7)	
Administration	85 (19.5)	37 (7.4)	122 (13.1)	
Hospital type				
General Hospitals urban	124 (28.5)	286 (57.5)	410 (44.0)	< 0.001
General Hospitals rural	103 (23.7)	59 (11.9)	162 (17.4)	
Tertiary Hospitals	208 (47.8)	152 (30.6)	360 (38.6)	
Job role				
Clinical work	175 (40.2)	268 (53.9)	443 (47.5)	< 0.001
Non-clinical work	138 (31.7)	103 (20.7)	241 (25.9)	
Both	122 (28.0)	126 (25.4)	248 (26.6)	
Work schedule				
Day shift	253 (58.2)	280 (56.3)	533 (57.2)	0.041
Night shift	24 (5.5)	13 (2.6)	37 (4.0)	
Day and night	158 (36.3)	204 (41.0)	362 (38.8)	
Type of work				
Full time	425 (97.7)	469 (94.4)	894 (95.9)	0.010
Part time	10 (2.3)	28 (5.6)	38 (4.1)	
Job contract				
Civil services	223 (51.3)	266 (53.5)	489 (52.5)	0.491
Yearly contract	212 (48.7)	231 (46.5)	443 (47.5)	
Current position				
Staff	339 (77.9)	419 (84.3)	758 (81.3)	0.001
Managerial	38 (8.7)	16 (3.2)	54 (5.8)	
Both	58 (13.3)	62 (12.5)	120 (12.9)	

Table 2 shows that job satisfaction was significantly higher among males (3.90 ± 0.762, *p* < 0.001), non- Saudi (4.12 ± 0.777, *p* < 0.001), those with older age ≥ 50 (4.06 ± 0.835, *p* < 0.001), those holding diploma’s degree (3.92 ± 0.744, *p* = 0.006), those being married (3.87 ± 0.771, *p* < 0.001), ≥ 15 years’ experience in their profession (4.00 ± 0.811, *p* < 0.001), working in tertiary hospitals (3.89 ± 0.796, *p* = 0.002), and those who work in the day shift (3.89 ± 0.741, *p* < 0.001). The mean score for each domain was obtained by dividing the sum of the responses on each question by four (the number of questions).

**Table 2:** Differences in the mean level of job satisfaction among HCWs according to their characteristics

Items	Mean	SD	F/t	*p* value
Sex*				
Male	3.90	0.762	3.866	< 0.001
Female	3.71	0.753		
Nationality*				
Saudi	3.75	0.750	-4.911	< 0.001
Non-Saudi	4.12	0.777		
Age categories***				
< 30 years	3.68	0.738	7.453	< 0.001
30 to < 40 years	3.73	0.714		
40 to < 50 years	3.90	0.808		
≥ 50 years	4.06	0.835		
Educational level***				
Diploma	3.92	0.744	5.187	0.006
Bachelor	3.74	0.756		
Postgraduate	3.79	0.800		
Marital status***				
Single	3.67	0.740	6.974	< 0.001
Married	3.87	0.771		
Divorced/widowed	3.68	0.698		
Monthly income (Saudi Riyal)***				
< 5000	3.94	0.667	1.837	0.139
5000 to < 10,000	3.80	0.759		
10,000 to < 25,000	3.80	0.770		
≥ 25,000	3.58	0.781		
Years of experience***				
< 5 years	3.64	0.726	11.379	< 0.001
5 to < 10 years	3.75	0.634		
10 to < 15 years	3.70	0.772		
≥ 15 years	4.00	0.811		
Profession***				
Physician	3.73	0.955	0.406	0.845
Nurse and Midwife	3.79	0.754		
Pharmacist	3.92	0.659		
Dentist	3.79	0.648		
Allied	3.82	0.761		
Administration	3.83	0.673		
Hospitals type***				
General (urban)	3.70	0.701	6.242	0.002
General (rural)	3.84	0.811		
Tertiary	3.89	0.796		
Job role***				
Clinical work	3.80	0.811	0.018	0.982
Non-clinical work	3.79	0.697		
Both	3.80	0.738		
Work schedule***				
Day shift	3.89	0.741	9.567	< 0.001
Night shift	3.67	0.812		
Day and night	3.68	0.771		
Type of work*				
Full time	3.79	0.765	-1.166	0.122
Part time	3.94	0.708		
Job contract*				
Civil services	3.79	0.765	-0.438	0.331
Yearly contract	3.81	0.761		
Current position***				
Staff	3.81	0.778	0.507	0.602
Managerial	3.83	0.672		
Both	3.74	0.705		

*(t) Independent sample t-test

***(F) One-way ANOVA

The multiple linear regression in Table 3 shows that the independent significant predictors of job satisfaction were sex, nationality, work schedule, and type of work (part-time job). Being female (B = − 0.198, *p* < 0.001), working at night and rotating shifts (B = − 0.318, *p* = 0.012; B = − 0.243, *p* < 0.001) were negatively associated with job satisfaction. On the other hand, being non-Saudi (B = 0.513, *p* < 0.001) and working in a part-time job (B = 0.247, *p* = 0.048) were positively associated with job satisfaction.

**Table 3:** Regression for the predictors of job satisfaction among HCWs in Jeddah province, Saudi Arabia

Variable	B	95% CI
**Sex** (male ref)		
Female	− 0.198***	− 0.310; − 0.086
Nationality (Saudi ref)		
Non-Saudi	0.513***	0.297; 0.730
Age (< 30 ref)		
30 to < 40 years old	− 0.059	− 0.243; 0.124
40 to < 50 years old	− 0.047	− 0.262; 0.168
≥ 50 years old	0.020	− 0.233; 0.273
Educational level (diploma ref)		
Bachelor	− 0.119	− 0.240; 0.001
Postgraduate	− 0.108	− 0.273; 0.056
Marital status (single)		
Married	0.048	− 0.075; 0.172
Divorced/widowed	− 0.045	− 0.250; 0.161
Work schedule (day shift ref)		
Night shift	− 0.318**	− 0.566; − 0.070
Day and night	− 0.243***	− 0.352; − 0.134
Hospitals type [general (urban) ref]		
General (rural)	− 0.021	− 0.175; 0.132
Tertiary	0.101	− 0.010; 0.212
Years of experience (< 5 years)		
5 to < 10 years	0.069	− 0.105; 0.243
10 to < 15 years	− 0.012	− 0.195; 0.171
≥ 15 years	0.144	− 0.056; 0.344
Monthly income (Saudi Riyal) (< 5000 SR ref)		
5000 to < 10,000	− 0.130	− 0.351; 0.092
10,000 to < 25,000	− 0.102	− 0.340; 0.137
≥ 25,000	− 0.306	− 0.657; 0.045
Profession (physician ref)		
Nurse and Midwife	0.092	− 0.104; 0.288
Pharmacist	0.192	− 0.120; 0.505
Dentist	0.220	− 0.211; 0.650
Allied	0.143	− 0.067; 0.353
Administration	0.113	− 0.145; 0.372
Job role (clinical work ref)		
Non-clinical work	− 0.044	− 0.190; 0.103
Both	− 0.006	− 0.131; 0.120
Type of work (full time ref)		
Part time	0.247**	0.002; 0.491
Job contract (civil services ref)		
Yearly contract	− 0.015	− 0.122; 0.093
Current position (staff ref)		
Managerial	− 0.056	− 0.269; 0.156
Both	− 0.060	− 0.213; 0.093
Constant	3.973***	3.642; 4.305
Number of observations		
Adjusted R^2^	0.092	
VIF (max)	6.246	

< 0.01 = ***, < *0.05* = **

The adjusted R^2^ of 0.092 indicates that approximately 9.2% of the variance in job satisfaction is explained by the predictors in the model after adjusting for the number of variables included. The VIF (max) was 6.246 < 10, which means there is no high multicollinearity between variables.

## Discussion

According to Herzberg’s theory, the motivation factors (intrinsic) contribute to job satisfaction, while the hygiene factors (extrinsic) decrease or prevent job dissatisfaction. The intrinsic factors include advancement and personal growth in the workplace. On the other hand, the extrinsic factors include salary, working conditions, policies and administration [30]. The data analysis revealed that the factors influencing HCWs’ job satisfaction are sex, nationality, work schedule, and type of work.

To our knowledge, this is the first study conducted in Saudi Arabia that includes HCWs in hospitals after completing the first phase of healthcare transformation in 2023 [20]. This transformation led to restructuring HCWs’ job contracts, which may affect their job satisfaction.

Male HCWs showed higher job satisfaction than females, aligning with previous studies conducted in India and Saudi Arabia, which attributed the differences between male and female HCWs to the variation in the harmony between societal, job roles, and cultural norms, such as household responsibilities, childcare, pregnancy-related challenges, and gender discrimination [31–34]. Females face obstacles in the conflict between demanding job roles (like night shifts), lack of professional development opportunities, and family obligations, resulting in lower job satisfaction [34, 35]. Meanwhile, males may regard their healthcare duties as fulfilling societal expectations; the work environment and support structure favour males. In addition, leadership roles are mostly occupied by males [34, 36], offering recognition that enhancing job satisfaction [37]. To bridge this gender gap, the Saudi government has incorporated women's empowerment into its national development plans, aiming to enhance their participation and provide greater leadership opportunities [34].

Non-Saudis reported higher job satisfaction than Saudis, aligning with an earlier study conducted in Saudi Arabia [38]. It linked this trend to better salaries, availability of technological tools, and high-quality facilities accessible to expatriates [38]. Healthcare workers from abroad receive salaries up to four times greater than those in their native countries, making the healthcare environment in Saudi Arabia attractive [39]. Similarly, a study in Oman emphasized the importance of economic incentives as a key factor in promoting expatriates' job satisfaction [40].

Conversely, other studies revealed higher job satisfaction among Saudis, highlighting factors like work-life balance, attractive salaries, and holiday benefits [11, 41]. These varied findings shed light on the complexity of determinants, suggesting that financial incentives alone do not fully explain job satisfaction, indicating that additional factors like job stability and cultural adjustment might also influence job satisfaction [17, 42]. This controversy asserts the complex nature of job satisfaction, and in light of Herzberg's theory, nationality influences both extrinsic (e.g., salary and resources) and intrinsic (e.g., work-life balance) factors [9].

Working on a day shift was linked with higher satisfaction. Our findings match previous studies in Iran and Palestine, which attributed improper day sleeping to health and well-being [43, 44]. This attribution is explained by the disturbed circadian rhythms of sleeping patterns as an aspect of our physiology [45].

Working in a part-time job indicates higher job satisfaction. This finding may be attributed to the Ministry of Health (MOH) regulation established in 2022 in Saudi Arabia, which allows healthcare professionals in public hospitals to work in the private sector for extra income after their regular working hours [46]. A study in Bangladesh revealed that work flexibility, such as a part-time job, can enhance the work-life balance among employees, which leads to overall life satisfaction [47].

## Study limitations

This study has limitations. First, using cross-sectional data may restrict the causal interpretation, as it only captures information at a single point in time; this lack of temporality compromises the inferential causal relationship interpretation. Second, the study was conducted in MOH-affiliated hospitals in the Jeddah health region, which limits the generalisability to all other types of hospitals (private, military, and university). A self-administered questionnaire may introduce response and social desirability biases, affecting the accuracy of reported satisfaction levels. Future studies can use a qualitative method to identify new determinants influencing the participants' job satisfaction level, which may not have been investigated earlier.

## Conclusion and policy implications

Job satisfaction was higher among non-Saudis and those working part-time jobs. On the other hand, it was lower in females and those working at night or rotating shifts. Based on Vision 2030, these findings can help policymakers plan a comprehensive strategy to foster job satisfaction among HCWs and ensure workforce stability. In addition, the working schedule should be reviewed by generating flexible shift times and increasing the incentives.

## Data Availability

Data available on request.
